# Ticagrelor is more effective than clopidogrel in carrier of nonfunctional *CYP2C19* allele who has diabetes and acute coronary syndrome - case report and literature review

**DOI:** 10.3934/molsci.2022004

**Published:** 2022-04-28

**Authors:** Rahel Tekeste, Gregorio Garza, Song Han, Jianli Dong

**Affiliations:** 1 School of Medicine, University of Texas Medical Branch, Galveston, TX, USA; 2 Health Sciences Division, University of Monterrey, Monterrey, NL, Mexico; 3 Department of Pathology, University of Texas Medical Branch, Galveston, TX, USA

**Keywords:** *CYP2C19*, clopidogrel, *P2RY12*, pharmacogenetics, intermediate *CYP2C19* metabolizer, MACE, ACS, PCI

## Abstract

Clopidogrel is a purinergic receptor *P2Y12* (*P2RY12*)-blocking pro-drug used to inhibit platelet aggregation in patients at risk for major adverse cardiac events (MACE), such as coronary artery disease and stroke. Despite clopidogrel therapy, some patients may still present with recurrent cardiovascular events. One possible cause of recurrence are variants in the cytochrome P450 2C19 (*CYP2C19*) gene. *CYP2C19* is responsible for the metabolism of many drugs including clopidogrel. Recent studies have associated pharmacogenetics testing of *CYP2C19* variants to guide clopidogrel therapy with a decreased risk of certain recurrent MACEs. Through a different mechanism, diabetes mellitus (DM) and obesity are also associated with clopidogrel treatment failure. We describe the case of a 64-year-old Caucasian woman with a history of acute coronary syndrome (ACS) and percutaneous coronary intervention (PCI), and DM/obesity, who presented to University of Texas Medical Branch (UTMB) in 2019 with a transient ischemic attack (TIA) while on clopidogrel/aspirin dual anti-platelet therapy. After *CYP2C19* genetic testing revealed that she was an intermediate metabolizer with a heterozygous *2 genotype, ticagrelor replaced the clopidogrel treatment regimen. No future MACEs were documented in the two-year patient follow-up. Thus, ACS patients with DM/obesity who have undergone PCI and are intermediate *CYP2C19* metabolizers may yield better treatment outcomes if prescribed ticagrelor instead of clopidogrel. Whether this improvement was due to genotype-guided therapy or the differing interactions of clopidogrel/ticagrelor in DM/obese patients is unknown based on available data. Regardless, *CYP2C19* genotype-guided treatment of ACS/PCI patients, with consideration of DM/obesity status, may provide effective individualized therapy compared to standard treatment. The inclusion of DM/obesity in this study is clinically relevant because DM/obesity has become a major health issue in the United States and worldwide.

## 1. Introduction

Major adverse cardiac events (MACE), such as coronary artery disease and stroke, are the leading cause of death in the United States [1]. Standard of care for patients with acute coronary syndrome, carotid artery stenosis, and ischemic heart disease includes percutaneous coronary intervention (PCI) and administration of dual antiplatelet therapy (aspirin and *P2RY12*-blocking drugs like clopidogrel). Goals of therapy include treatment and prevention of vaso-occlusive events and thrombotic complications. Clopidogrel, a *P2RY12*-blocking prodrug, is metabolized by cytochrome P450 (*CYP450*) liver enzymes including *CYP2C19*. *CYP2C19* is involved in the phase I metabolism of medications including clopidogrel, proton-pump inhibitors, and some selective serotonin reuptake inhibitors. *CYP2C19* is one of the most frequently tested pharmacogenomics genes in clinical laboratories and is included in the Food and Drug Administration (FDA) Table of Pharmacogenomic Biomarkers in Drug Labeling for several FDA-approved drugs [2]. In hepatocytes, *CYP2C19* metabolizes clopidogrel into 2-oxo-clopidogrel, and then into its active form [3]. Once active, clopidogrel irreversibly inhibits normal functioning of the given platelet for the rest of its lifespan (7–10 days). Thus, discontinuation of clopidogrel administration results in a return to baseline platelet function upon subsequent platelet turnover [4].

**Figure 1.**
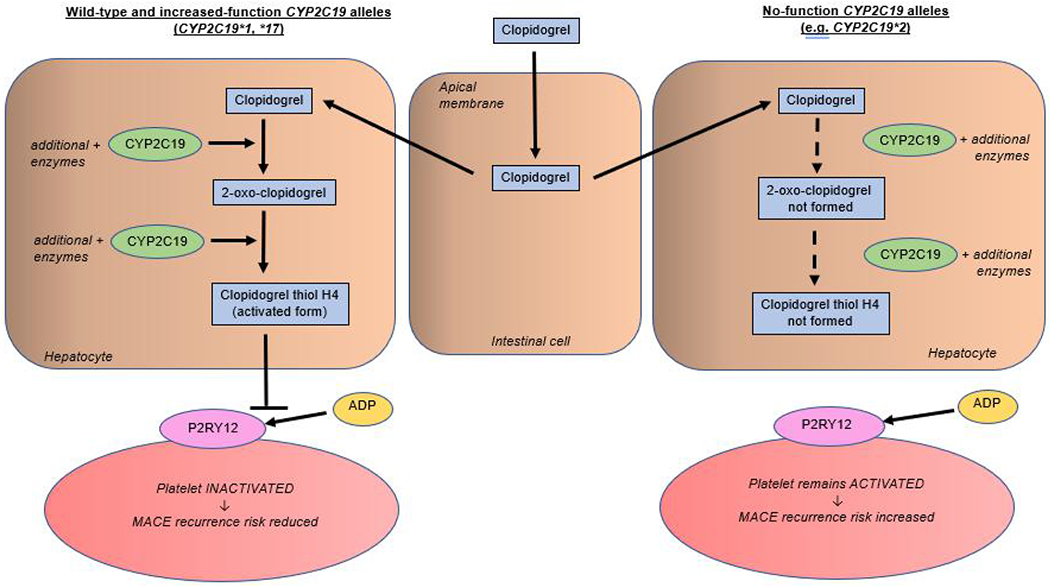
Comparison of wild-type and increased-function (*1 and *17) and no-function (e.g. *2) *CYP2C19* alleles on clopidogrel activation, and subsequently platelet activation.

The *CYP2C19* gene is located on chromosome 10q23.33 and is highly polymorphic with approximately 39 star (*) alleles, leading to possible variants in *CYP2C19* enzyme activity and subsequent altered drug response [5,6]. For clopidogrel and *CYP2C19*, *1 is the normal function allele. Individuals homozygous for *1 are normal metabolizers with normal platelet inhibition and normal residual platelet aggregation. The *17 is an increased function allele. Individuals with one or two copies of *17 allele are ultra-rapid metabolizers with increased platelet inhibition and decreased residual platelet aggregation. The *2, *3, *4, *5, *6, *7, and *8 are no-function alleles. Individuals with one or two no-function allele are intermediate and poor metabolizers, respectively. These individuals have higher risk of reduced platelet inhibition, increased residual platelet aggregation, and adverse cardiovascular events; alternative antiplatelet therapy (e.g., ticagrelor) is indicated if there are no contraindications [7,8], Figure 1 summarizes the effects of wild type versus no-function *CYP2C19* alleles on clopidogrel metabolism and platelet activation.

The left side of the Figure 1 illustrates the proper activation of clopidogrel in the presence of *1 or *17 allele. Once activated, clopidogrel prevents platelet activation by blocking adenosine diphosphate (ADP) binding onto the *P2RY12* platelet receptor. Inactivated platelets cannot properly clot. Thus, *P2RY12*-blockers like clopidogrel help prevent future major adverse cardiac events (MACE). The right side of the figure illustrates the impaired activation of clopidogrel in the presence of no-function *CYP2C19* like *2 allele. Without activated clopidogrel, ADP receptor binding is unimpeded, leading to excessive platelet activation and increased MACE recurrence risk [3,44].

**Figure 2.**
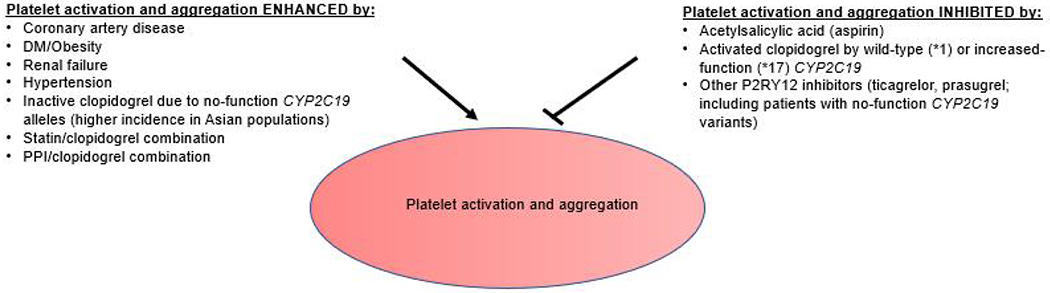
Summary of factors affecting platelet activation and aggregation.

Given that the efficacy of clopidogrel is, in part, dependent upon it being metabolized by *CYP2C19* to an active metabolite, intermediate and poor metabolizers can have reduced antiplatelet responses when treated with clopidogrel. Based on this information, the FDA issued a boxed drug label warning indicating potential for reduced efficacy (increased adverse cardiovascular outcomes) in March 2010. This warning demonstrated the importance of genotypic determination of the patients who are taking clopidogrel [9]. Previous studies have shown that *CYP2C19* no-function variants lead to an increased risk of cardiovascular events. However, evidence supporting whether genotype-guided treatment is superior to current standard therapy in lowering MACE recurrence risk (including in ACS patients) varies based on what the given study defines as standard or genotype-guided treatment [10–14]. The current study defines standard therapy as administration of clopidogrel without *CYP2C19* genotyping. Other factors aside from *CYP2C19* genotype have been hypothesized to have an inhibitory effect on clopidogrel metabolism or platelet function. These factors include certain comorbidities (DM, obesity, hypertension, and renal failure), medications (proton-pump inhibitor and statin use), and demographics (e.g., Asian heritage). In addition, coronary artery disease, which itself is affected by heperlipidemia, hypertension, and diabetes, has also been associated with enhanced platelet activation [15–18]. Figure 2 summarizes these risk factors. Therefore, it appears that a patient’s response to clopidogrel is multifactorial.

We describe the case of a 64-year-old Caucasian woman with a history of acute coronary syndrome (ACS) and percutaneous coronary intervention (PCI), and DM/obesity, who presented to University of Texas Medical Branch (UTMB) in 2019 with a transient ischemic attack (TIA), while on clopidogrel/aspirin dual anti-platelet therapy. After *CYP2C19* genetic testing revealed that she was an intermediate metabolizer with a heterozygous *2 genotype, ticagrelor replaced the clopidogrel treatment regimen. No future MACEs were documented in the two-year patient follow-up. Thus, acute coronary syndrome (ACS) patients with DM/obesity who have undergone PCI and are intermediate *CYP2C19* metabolizers may yield better treatment outcomes if prescribed ticagrelor instead of clopidogrel. Whether this improvement was due to genotype-guided therapy or the differing interactions of clopidogrel/ticagrelor in DM/obese patients is unknown based on available data. Regardless, *CYP2C19* genotype-guided treatment of ACS/PCI patients, with consideration of DM/obesity status, may provide effective individualized therapy compared to standard treatment. The inclusion of DM/obesity in this study is clinically relevant because DM/obesity has become a major health issue in the United States and worldwide.

Factors of Figure 2 include salicylic acid hydrolyzed from aspirin, *P2RY12* inhibiting drugs, *CYP2C19* alleles that affect clopidogrel activation, certain comorbidities (DM, obesity, renal failure, and hypertension), medications (proton-pump inhibitor and statins).

## 2. Clinical history

In 2013, a 58 year-old Caucasian woman was admitted to UTMB with symptoms of facial numbness, headache, and chest pain, and was diagnosed with a transient ischemic attack (TIA) and coronary artery disease (CAD). The patient had been treated for type 2 diabetes mellitus, hyperlipidemia, and hypertension at UTMB since 2007. For these conditions, she had taken metformin HCl, enalapril, maleate, pioglitazone, atorvastatin, and hydrochlorothiazide (HCTZ). In general, the patient appears to have been compliant with her medication regimen throughout the breadth of this study. Her BMI was 33.79 (30–39.9) kg/m^2^, classifying her as obese. She denied history of smoking or alcohol use. She denied history of renal failure or proton-pump inhibitor use. She denied history of smoking or alcohol use. Table 1 shows key anthropometric and hematologic parameters for this patient.

She was admitted to UTMB for an elective left heart catheterization and PCI. It was in this encounter that she was first prescribed 75 mg clopidogrel and low-dose aspirin. The patient stayed on clopidogrel throughout 2013. However, in 2014, she stopped taking clopidogrel because she was unable to afford the medication. She was kept on aspirin since 2013.

In March 2016, she was admitted to UTMB for a severe cerebellar stroke. A vascular sonography visit revealed that she had bilateral carotid bruits. She received a coronary artery bypass graft (CABG) with left internal mammary artery to left anterior descending artery, and saphenous vein graft to the obtuse marginal artery. She was prescribed clopidogrel and continued on low-dose aspirin. In January 2018, the patient received a left circumflex and right coronary PCI with drug-eluting stent. She was discharged on ticagrelor 90 mg and low-dose aspirin. However, after a few months, the patient stopped taking ticagrelor because she was no longer able to afford it. She switched back to clopidogrel and aspirin.

In January 2019, the patient presented to the UTMB emergency department with symptoms of transient ischemic attack (TIA). Neurologists requested genetic testing for *CYP2C19*. Results revealed a *1/*2 genotype, thus exhibiting intermediate metabolism of clopidogrel. Clopidogrel was replaced with ticagrelor 90 mg. UTMB social worker searched for a discounted version of ticagrelor for the patient.

## 3. Patient follow-up

Future encounters, as indicated on patient electronic medical records (last accessed in 4/10/2022), have been unremarkable from a cardiac standpoint for the next three years. No cardiovascular-related ER visits were documented, and no bleeding and/or associated disorders have been recorded. Ticagrelor prescription has been continued since her 2019 ER visit.

## 4. Laboratory role in diagnosis

*CYP2C19* genetic testing was performed in the Molecular Diagnostics Laboratory at UTMB in Galveston, Texas. Genomic DNA was extracted and purified from whole blood using QIAamp DNA Blood Mini kit (Qiagen Inc., Germantown, MD). Following polymerase chain reaction (PCR) and exonuclease digestion, *CYP2C19* genotyping was performed to detect *1, *2, *3, *4, *6, *8, *9, *10, and *17 alleles using eSensor *CYP2C19* Genotyping Test on XT-8 according to manufacturer’s instructions (GenMark Diagnostics, Inc., Carlsbad, CA).

## 5. Discussion

Dual antiplatelet therapy (DAPT), a combination of aspirin and a *CYP2C19* inhibitor, has been shown to reduce MACE recurrence in patients with ACS or patients undergoing PCI. Reduction in MACE recurrence is at the expense of increased risk of major bleeding, as compared with aspirin monotherapy. The clinical utility of *CYP2C19* genotype testing in the treatment of ACS/PCI patients on clopidogrel is still under study. The 2011 Clinical Pharmacogenetics Implementation Consortium guideline (CPIC), along with a 2013 update, recommend it as standard clinical test. The CPIC guideline, along with the US Food and Drug Administration’s (FDA) Boxed Warning, recommend that poor metabolizers be switched to an alternative therapy like ticagrelor or prasugrel, as long as the medication is not contraindicated clinically [7,9]. Such recommendations have also been incorporated into the American College of Cardiology/American Heart Association Guidelines regarding the DAPT for ACS patients [19].

Our patient’s genotypical category as an intermediate metabolizer of clopidogrel may have attributed to her history of recurrent cardiovascular events, despite being on clopidogrel. The lack of reported cardiovascular events on patient follow up, after switching to ticagrelor, further strengthens the hypothesis of a better clinical outcome with the new drug administration. It should be noted that the CPIC guideline does not take into account DM/obesity when determining clinical utility of *CYP2C19* genetic testing.

In addition, the CPIC guideline 2013 update states that doubling clopidogrel dosage improved platelet inhibition in *2 heterozygote patients. However, *2 homozygous patients were not found to experience the same degree of platelet inhibition. CPIC also concludes no change in the rate of MACE recurrence for *2 heterozygotes [7]. These findings explain why increasing clopidogrel dosage is not recommended by the CPIC, and thus was not presented in our patient’s genetic panel results as a therapeutic recommendation.

Ticagrelor and prasugrel are viable alternatives to clopidogrel treatment failure because they are also P2RY12 inhibitors. However, with a different metabolism than clopidogrel, ticagrelor and prasugrel are metabolized by different *CYP2C19* enzymes, and are thus not affected by *CYP2C19* variants [20,21].

The relative functionalities of ticagrelor and prasugrel in reducing thrombosis also differ from that of clopidogrel. A 2020 systematic review found that usage of ticagrelor or prasugrel significantly reduced the risk of recurrent MACEs in ACS/PCI patients with no-function *CYP2C19* variants when compared to clopidogrel [22]. In addition, ticagrelor has been associated with more consistent inhibition of platelet aggregation and a greater reduction in ischemic events in ACS patients compared to clopidogrel, regardless of the presence of diabetes [23]. The role of diabetes in the pathophysiology of our patient’s clopidogrel treatment failure is discussed further in this paper.

Not all studies have identified a link between ticagrelor or prasugrel administration and decreased MACE recurrence compared to clopidogrel. A 2020 cohort study by Turgeon et al. [24] found no significant MACE reduction in ACS/PCI patients when administering ticagrelor compared to clopidogrel. The authors attribute these findings to differences in methodology, patient population, and improved interventional cardiology procedures as compared to previous studies [24–26].

Despite the possible therapeutic benefits of ticagrelor and prasugrel, these medications come at a higher risk of bleeding when compared to clopidogrel [25,27]. The increased risk of bleeding associated with ticagrelor may be of concern for our patient due to her history of mild anemia. Anemia is defined by the World Health Organization as having hemoglobin <13 g/dL in men and <12 g/dL in women [28]. Anemic patients post-PCI are at a significant risk of bleeding, according to the Academic Research Consortium for High Bleeding Risk (ARC-HBR) [29]. Post-PCI, our patient’s recorded hemoglobin levels were 10.3–11.8 g/dL, aside from a post-operative anemia from her CABG, which was treated with a blood transfusion. Since she has only presented mild anemia after being placed on ticagrelor, we believe that her risk of future bleeding while on ticagrelor is not as significant as is described in literature. However, close follow-up is essential and is being done as of the time of publishing. Because of this risk of bleeding, the CPIC highly recommends considering both clinical history and *CYP2C19* genotyping when establishing a treatment plan. This allows for appropriate individualized therapy [30].

The CPIC also identifies the clinical scenarios in which *CYP2C19* testing should be ordered. Testing may be useful for all patients who undergo PCI, or for those with high risk of poor outcomes while on suboptimal platelet therapy. The later includes patients with high-risk cardiovascular clinical features such as DM and ACS, as is the case for our patient. Genotype testing takes several hours to perform [31]. Thus, the CPIC guideline highly recommends ordering testing as soon as possible, although that may not be feasible in an acute setting like ACS [30].

In addition to *CYP2C19* genotype, comorbidities such as diabetes and obesity are important factors in determining appropriate anti-platelet therapy [36–38]. Diabetes and obesity may have an additive effect along with no-function *CYP2C19* genotypes on clopidogrel treatment failure. DM-associated clopidogrel treatment failure may be attributed to insulin resistance, resulting in increased platelet reactivity compared to non-DM patients. This is because insulin has been found to inhibit the increased platelet calcium levels and suppressed cyclic AMP (cAMP) required for proper platelet activation. Insulin resistance has been linked with increased platelet activity, and increased risk of cardiovascular-related sequelae. Treating DM patients with no-function *CYP2C19* genotypes may thus involve a higher degree of platelet inhibition than treating non-DM patients [32–34]. Patients with a BMI ≥25kg/m^2^ likewise have been associated with decreased platelet inhibition [35]. Therefore, the role of BMI in the effectiveness of clopidogrel therapy also has clinical relevance. One study showed that patients with elevated BMI (most of which > 28.3) were more likely to exhibit elevated platelet reactivity and increased thrombus risk, despite being on clopidogrel and aspirin. This relationship was not found in patients administered ticagrelor and aspirin [36]. Our patient’s BMI is 33.79. Thus, her BMI, in addition to her DM status, could influence her clopidogrel treatment failure in addition to her no-function *CYP2C19* genotype. Studies like this may explain why ticagrelor was associated with fewer instances of MACEs in our patient compared to clopidogrel.

The combined role of DM/obesity and no-function *CYP2C19* genotype in clopidogrel treatment failure was also exhibited in a 2020 Egyptian randomized trial. This study analyzed the roles of DM, *CYP2C19* genotype, and antiplatelet therapy type (clopidogrel or ticagrelor) in platelet aggregation and MACE recurrence. The study found that patients who had diabetes and no-function *CYP2C19* variants had the highest incidence of recurrent ACS and maximum platelet aggregation when administered clopidogrel compared to all other groups [39]. Thus, increased ACS recurrence may be due to clopidogrel treatment failure associated with both DM and a no-function *CYP2C19* genotype. Other studies have also identified a possible association between BMI and no-function *CYP2C19* genotype as an influence on clopidogrel treatment failure [40].

Not all studies have identified a significant difference in utilizing genotype-guided therapy (ticagrelor/prasugrel administration in no-function *CYP2C19* variants) compared to conventional therapy (clopidogrel administration without *CYP2C19* testing) in reducing risk of MACEs. For example, a 2020 TAILOR-PCI Randomized Clinical Trial found that incidence of cardiovascular death and recurrent ischemia was not significantly changed by the use of genotype-guided versus conventional therapy in a 12-month period [13]. A diabetic subset of the patients receiving genotype-guided therapy (15 out of 253, or 6.2% of total participants) likewise did not experience a statistically significant reduction in MACEs compared to those receiving conventional therapy (22 out of 257, or 8.8% of total participants). Recorded BMI in the study ranged from 24.3–31.9, depending on the treatment group. This excludes our patient’s BMI of 33.79. We were not aware of studies that further stratified BMI beyond comparing a patient’s weight to the normal 18-25kg/m^2^ range. Thus, the clinical significance of our patient’s BMI relative to those in the TAILOR-PCI study is unknown. Since diabetic/obese patients were not the focus of the Pereira study, and that a relatively small number of these participants were evaluated compared to the study’s total, the impact of these findings on our present study is unknown. Future randomized trials may help elucidate the effects of diabetic status and BMI on the clinical utility of genotype-guided therapy.

Additional studies that failed to demonstrate the clinical utility of *CYP2C19* testing are illustrated in a 2018 review by Zeb et al. [41]. This article discusses large studies that proposed that *CYP2C19* genotype was not a significant factor in determining clopidogrel treatment response. However, the article does promote utilization of *CYP2C19* testing in high-risk clinical scenarios. Our patient’s clopidogrel treatment failure may be due to a combination of impaired clopidogrel metabolism (due to presence of no-function *CYP2C19* variant) and decreased inhibition of platelet reactivity (possibly by DM and obesity). Whether genotype-guided or standard treatment is more contributory in reducing MACE recurrence is still unknown based on the available literature. Regardless, our study and literature review have shown that the administration of clopidogrel in intermediate metabolizing ACS/PCI patients with DM/obesity is not recommended. Ticagrelor is a viable alternative.

The administration of ticagrelor instead of clopidogrel in our patient’s treatment plan is significant, not only because ticagrelor is unaffected by *CYP2C19* variants, but also because of its increased effectiveness in treating patients with comorbidities in comparison to clopidogrel. Though not all studies evaluating the clinical utility of *CYP2C19* genotype testing consider DM or an elevated BMI, we highlight the importance of including such factors because of the possible association with recurrent MACEs. In addition, the above-mentioned studies, along with our findings, show the significance of utilizing both *CYP2C19* genotype and cardiovascular-related risk factors when establishing a treatment plan for patients with a history of recurrent MACEs. Lastly, acknowledgement of the decreased affordability of ticagrelor compared to clopidogrel is necessary in utilizing genotype-guided treatment. We found that discussing both the clinical and financial impact of prescribing ticagrelor instead of clopidogrel would be pertinent due to the stark price gap between the two drugs. According to a October 20, 2021 National Average Drug Acquisition Cost of the United States Centers for Medicare & Medicaid Services, clopidogrel 75 mg was $0.07 per day, and ticagrelor was $6.60 per day. Thus, a one-month supply of clopidogrel cost $2.10 and ticagrelor cost $198. This makes ticagrelor approximately 94 times more expensive than clopidogrel [42]. Thus, an important component of utilizing *CYP2C19* testing should be accounting for this increased price point. Providers should continue to help patients like the one in this study to afford ticagrelor if clinically indicated because of its increased clinical usefulness compared to clopidogrel [43]. Thus, we believe that a thorough review of literature, consideration of patient risk factors, and medication affordability is beneficial for adequate patient management. The cost of *CYP2C19* genetic testing itself is also clinically relevant. One study calculated the cost of testing as $250–$292 based on pricing data from Medicare. When taking into account the length and cost of treatment, implementing genetic testing was associated with decreased cost per quality-adjusted life years gained compared to therapy without genetic testing [44]. Thus, utilization of genetic testing for ACS/PCI patients could provide economic and clinical benefits.

Detailing our patient’s history of cardiovascular encounters at UTMB demonstrates the extent of her cardiovascular disease exacerbations while on clopidogrel. It also displays the clinical utility of *CYP2C19* genetic testing. If she was continued on clopidogrel and aspirin after her 2019 TIA, she may have continued to experience cardiovascular disease exacerbations and a general decline in health. Inclusion of an ACS patient with DM/obesity in our study reflects the increased prevalence of cardiovascular disease, DM, and obesity worldwide [45,46]. Thus, research such as ours may lead to more effective treatment recommendations for these populations. Further studies should utilize randomized controlled trials to study patients with DM/obesity and multiple MACE risk factors over a longer time period to provide a clearer perspective on the specific clinical utility of *CYP2C19* genetic testing in comparison to standard treatment.

## Figures and Tables

**Table 1.** Key anthropometric and hematologic parameters for the studied patient.

Parameters	2013	2019	2021/2022
BMI (kg/m2)	33.78	34.17	34.17
Height (ft)		5.0	
Weight (lb)	173	175	175
HbA1C (%)	6.8	7.7	8.1
Hemoglobin (g/dl)	12.2	11.1	12.4
LDL cholesterol (mg/dl)	135	53	45
HDL cholesterol (mg/dl)	60	47	41
